# Shattering the Glass Scalpel: Analyzing Female Representation in Orthopedic Surgery Across the United States

**DOI:** 10.7759/cureus.80088

**Published:** 2025-03-05

**Authors:** Rachel A Loyst, Sofia Hidalgo Perea, Taylor Vanhelmond, Diana Patterson

**Affiliations:** 1 Department of Orthopaedic Surgery, Renaissance School of Medicine at Stony Brook University, Stony Brook, USA; 2 Orthopaedic Surgery, Stony Brook University, Stony Brook, USA

**Keywords:** diversity, female, gender, orthopedic surgery, residents

## Abstract

Background

Orthopedic surgery has historically been a male-dominated field, with slower growth in female participation compared to other surgical specialties. The present study aims to assess the extent of this influence, examine whether the dynamic is changing, and further analyze female participation trends, focusing on various regions of the United States and including analyses of female residents.

Methodology

Orthopedic surgery residency programs were surveyed online. A list of 200 programs was sourced from the Association of American Medical Colleges, which provided details including the program names, cities, states, regions, and website links. The homepages of the residency program as well as the department of orthopedic surgery were surveyed for any mention of women or female in orthopedics, number of female residents, total number of residents (class of 2025-2029), number of female faculty operative orthopedic faculty, total number of operative orthopedic faculty physicians, specialty of the female orthopedic faculty, program director gender, assistant program director gender, and chair of the program gender. Data analyses were performed using SPSS Software version 28.0 (IBM Corp., Armonk, NY, USA) and Rstudio version 1.4.1106 (Boston, MA, USA).

Results

In total, 55 Midwest (28%), 53 Northeast (27%), 63 South (32%), and 27 West (14%) orthopedic surgery residency programs were included. Female orthopedic surgery residents comprised 23.7% of all orthopedic residents across the United States. When analyzed by geographic region, female residents comprised 22.7% of all residents in the Midwest, 24.3% in the Northeast, 22.5% in the South, and 27.0% in the West. In the Northeast region, there was a notable upward trend in the percentage of female residents across PGY5 to PGY1 years (τ = 1, p = 0.027). A total of 25 (12.6%) programs had a section in their residency program or orthopedic department website homepage that mentioned and/or was dedicated to women or females. There was a significant association between region and homepage mention of women (χ² (8, N = 198) = 105.7; p < 0.001). A total of 12.9% of all orthopedic residency program directors were female, while 21.2% of all associate program directors were female.

Conclusions

These findings underscore the importance of monitoring and addressing regional disparities in gender representation within orthopedic surgery residency programs. By identifying trends and disparities, healthcare institutions and policymakers can implement targeted interventions to promote gender equity and cultivate a diverse and inclusive workforce.

## Introduction

Orthopedic surgery is traditionally a male-dominated field, but this study aims to assess the extent of this influence and examine whether the dynamic is changing. Women comprise only 6% of orthopedic surgeons [1]. Compared to other surgical specialties, orthopedic surgery has experienced slower growth in female participation. A 2014 survey found that while 15% of medical students and 11% of interns were interested in orthopedic surgery, only 2% were women [2].

Key barriers identified in previous research include cultural and gender biases, lack of mentorship and role models, challenges in work-life balance, discrimination and harassment, and insufficient exposure and encouragement during training [1,3]. Regions with a stronger emphasis on diversity, equity, and inclusion (DEI) initiatives, potentially influenced by progressive academic cultures, state policies, or institutional missions, may foster a more supportive environment for female applicants. Despite a rise in female involvement, with female residents increasing from 11.0% in 2005-2006 to 14.0% in 2016-2017, orthopedic surgery still lags behind other specialties [4,5]. The present study aims to further analyze these trends, focusing on various regions of the United States and including analyses of female faculty as well as residents.

Efforts to address these disparities have included initiatives such as targeted mentorship programs, increased visibility of female orthopedic surgeons, and research examining the impact of institutional culture on gender diversity [6,7]. However, the extent to which these efforts have influenced recruitment and retention remains unclear. This study aims to provide an updated analysis of these trends, incorporating a regional perspective on female representation in both orthopedic residency and faculty positions. This study will review trends in female leadership roles or changes in female residency representation over time. By identifying patterns and disparities across different geographic areas, this research seeks to further contextualize ongoing challenges and potential solutions to fostering greater gender inclusivity in orthopedic surgery.

## Materials and methods

Study design

Orthopedic surgery residency programs were surveyed online. The list of 200 orthopedic surgery residency programs was sourced from the Association of American Medical Colleges (AAMC), which provided details including the program names, cities, states, regions, and website links. The AAMC divided the programs into four regions, namely, Midwest, Northeast, South, and West (Table 1). As this study used publicly available information, it was exempt from approval by our University’s Institutional Review Board.

**Table 1:** Regional state breakdown. Table credits: Sofia Hidalgo Perea.

Region	States included
Midwest	Illinois, Indiana, Iowa, Kansas, Ohio, Michigan, Minnesota, Missouri, Nebraska, North Dakota, South Dakota, and Wisconsin
Northeast	Connecticut, Maine, Massachusetts, New Hampshire, New Jersey, New York, Pennsylvania, Rhode Island, and Vermont
South	Alabama, Arkansas, Delaware, District of Columbia, Florida, Georgia, Kentucky, Louisiana, Maryland, Mississippi, North Carolina, Oklahoma, South Carolina, Tennessee, Texas, Virginia, and West Virginia
West	Alaska, Arizona, California, Colorado, Hawaii, Idaho, Montana, Nevada, New Mexico, Oregon, Utah, Washington, and Wyoming

From the original 200 programs surveyed from the AAMC website, two could not be found online and were therefore omitted from the statistical analysis. The homepages of the residency program as well as the department of orthopedic surgery were surveyed for any mention of women or female in orthopedics, number of female residents, total number of residents (class of 2025-2029), number of female faculty operative orthopedic faculty, total number of operative orthopedic faculty physicians, specialty of the female orthopedic faculty, program director gender, assistant program director gender, and chair of the program gender.

Inclusion and exclusion criteria

Orthopedic faculty included in this analysis included physicians who had successfully completed an orthopedic surgery residency (MD and DO degrees). Non-operative orthopedic faculty such as research staff, non-operative sports medicine, podiatrists, and support staff (physician assistants, physiatrists, sports medicine psychologists, athletic trainers) were excluded from the analysis. Programs that did not have their resident or faculty information available online were also omitted from those respective statistical analyses. For resident analysis, 13 programs were excluded, while 16 programs were excluded in the faculty analysis. During the analysis of leadership positions, four programs were excluded entirely due to missing all leadership data. For each specific leadership role, exclusions were made for programs where information was absent on their respective websites. Notably, 81 associate program director positions and 44 chair positions were additionally excluded. However, as many programs do not have associate program directors, the data analysis proceeded as planned. To enhance data reliability, two reviewers independently verified publicly available program information to minimize errors and ensure consistency. Additionally, discrepancies in reported faculty or leadership roles were cross-referenced with institutional directories when possible.

Statistical analysis

The data were collected and analyzed. Data analyses were performed using SPSS Software version 28.0 (IBM Corp., Armonk, NY, USA) and Rstudio version 1.4.1106 (Boston, MA, USA). The Mann-Kendall test was used to evaluate trends in the percentage of female residents across different regions from PGY5 to PGY1 years. A chi-square test was conducted to examine the relationship between region and homepage mention of women, region, and female leadership (program director, associate program director, and chair).

## Results

A total of 198 orthopedic surgery residency programs were included in this study, of which 55 (28%) were in the Midwest, 53 (27%) were in the Northeast, 63 (32%) were in the South, and 27 (14%) were in the West (Table 2).

**Table 2:** Demographic data of orthopedic residency programs included in this study. Statistical analyses were completed using the Mann-Kendall test.

Region	Programs (N = 198)	Representation of programs per region (%)	χ² (p-value)
Midwest	55	28	105.7 (<0.001)
Northeast	53	27	
South	63	32	
West	27	14	

**Figure 1:**
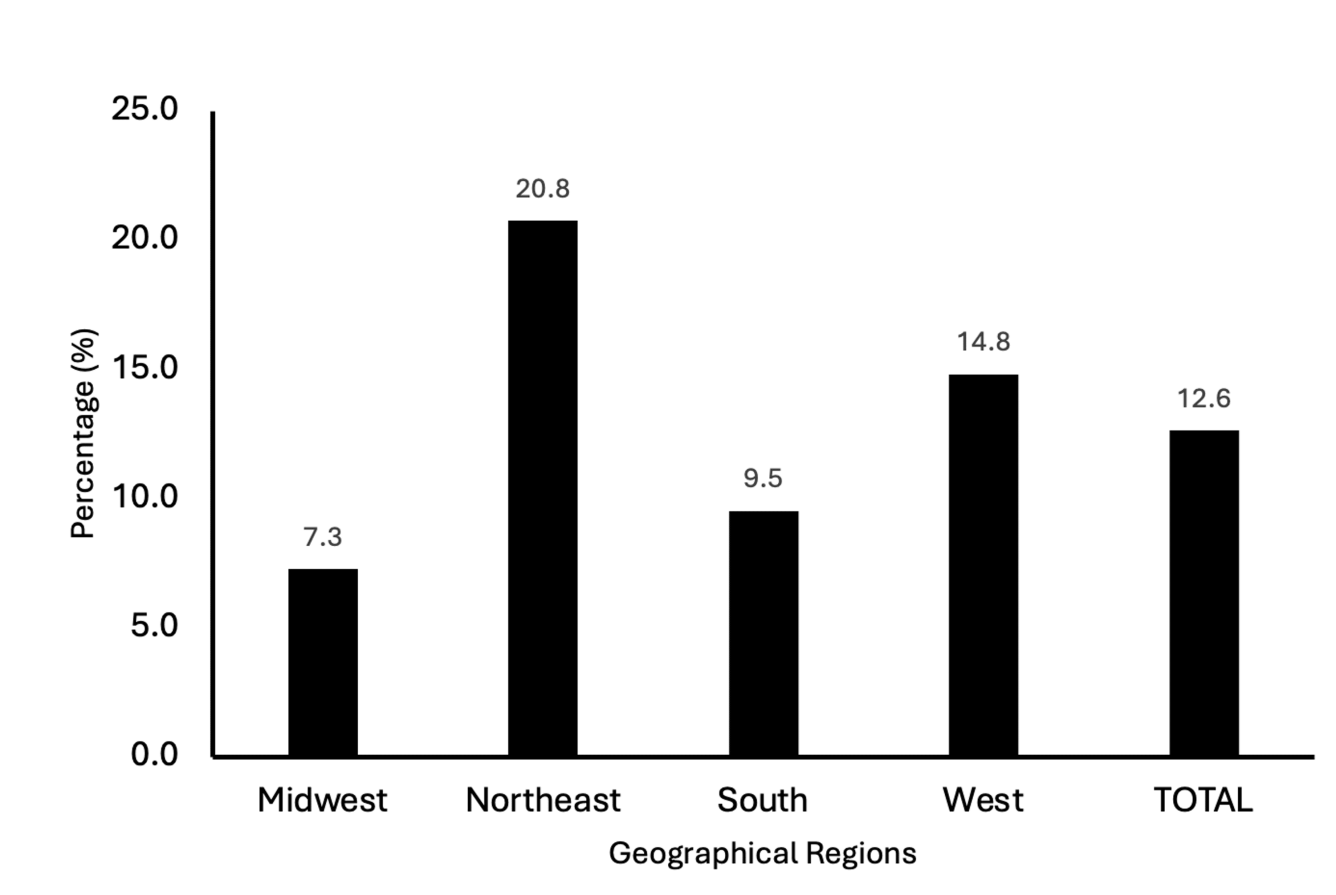
Regional number and proportion of programs with a homepage mention of women or female in orthopedics.

A total of 25 (12.6%) programs had a section in their residency program or orthopedic department homepage website that mentioned and/or was dedicated to women or females in orthopedics (Figure 1). There was a significant association between region and homepage mention of women (χ² (8, N = 198) = 105.7; p < 0.001). The Northeast had the greatest percent per region of programs that featured women on their homepage at 11 (20.8%) mentions, followed by the West at 4 (14.8%), South at 6 (9.5%), and Midwest at 4 (7.3%).

**Figure 2:**
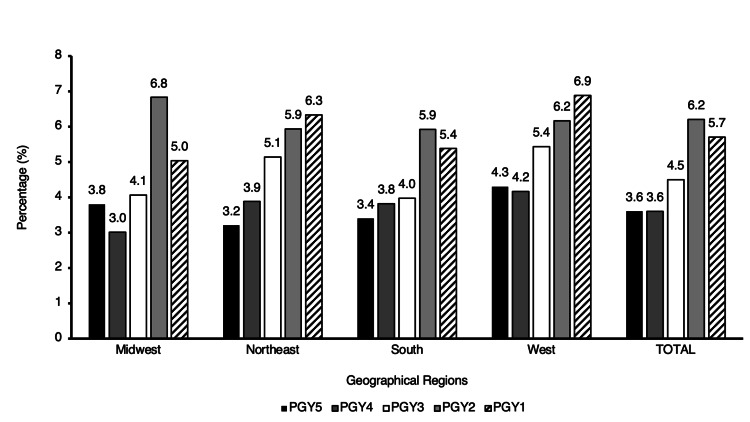
Regional representation of female residents per postgraduate year.

There are 1,026 female orthopedic surgery residents, which comprised 23.7% of all orthopedic residents across the United States. When analyzed by geographic region, there were 280 female residents, which comprised 22.7% of all residents in the Midwest, 308 (24.3%) in the Northeast, 289 (22.5%) in the South, and 149 (27.0%) in the West. When broken down by PGY, there were 249 female residents which constituted 5.7% of PGY1 orthopedic residents, 269 (6.2%) of PGY2, 196 (4.5%) of PGY3, 158 (3.6%) of PGY4, and 155 (3.6%) PGY5. In the PGY1 category, there were 62 female residents which constituted 5.0% of all residents in the Midwest, 80 (6.3%) in the Northeast, 69 (5.4%) in the South, and 38 (6.9%) in the West. For PGY2, there were 84 female residents, which made up 6.8% of all residents in the Midwest, 75 (5.9%) in the Northeast, 76 (5.9%) in the South, and 34 (6.2%) in the West. In the PGY3 category, there were 50 female residents which accounted for 4.1% of all residents in the Midwest, 65 (5.1%) in the Northeast, 51 (4.0%) in the South, and 30 (5.4%) in the West. For PGY4, there were 37 female residents which accounted for 3.0% of all residents in the Midwest, 49 (3.9%) in the Northeast, 49 (3.8%) in the South, and 23 (4.2%) in the West. Finally, for PGY5, 47 female residents constituted 3.8% of all residents in the Midwest, 40 (3.2%) in the Northeast, 44 (3.4%) in the South, and 24 (4.3%) in the West (Figure 2).

In the Northeast region, there was a notable upward trend in the percentage of female residents across PGY5 to PGY1 years (τ = 1, p = 0.027). Additionally, the South and West regions exhibited a moderate positive trend in female resident percentages, yet these trends did not reach statistical significance (τ = 0.8, p = 0.086; τ = 0.8, p = 0.086, respectively). Additionally, no significant trend was observed in the Midwest region (τ = 0.6, p = 0.22).

**Figure 3:**
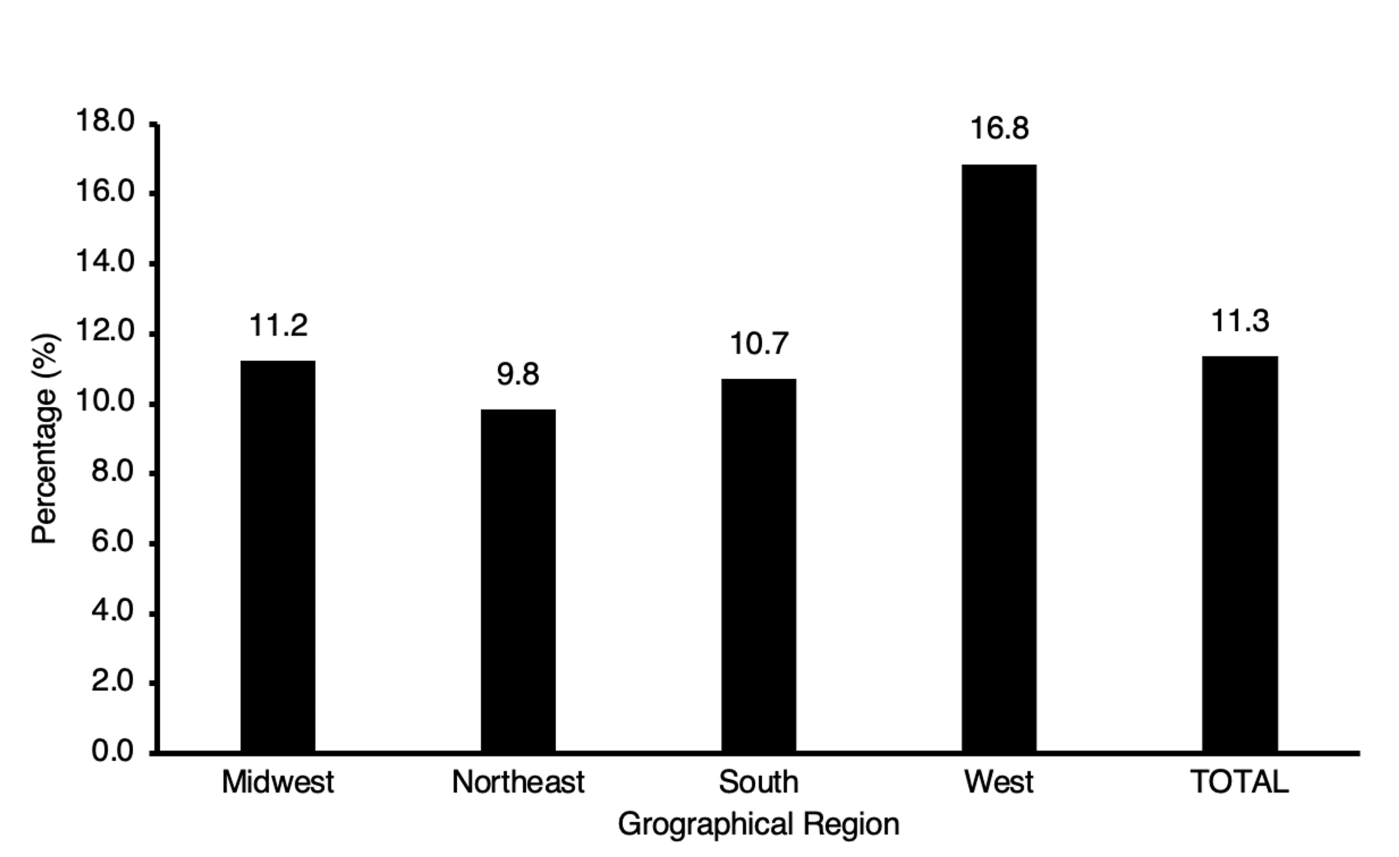
Regional representation of operative female orthopedic faculty.

A total of 730 (11.3%) of all operative orthopedic faculty was female across the different geographical regions presented in this study. In the Midwest, 181 female surgeons constituted 11.2% of operative orthopedic faculty, 200 (9.8%) in the Northeast, 210 (10.7%) in the South, and 139 (16.8%) in the West (Figure 3).

**Figure 4:**
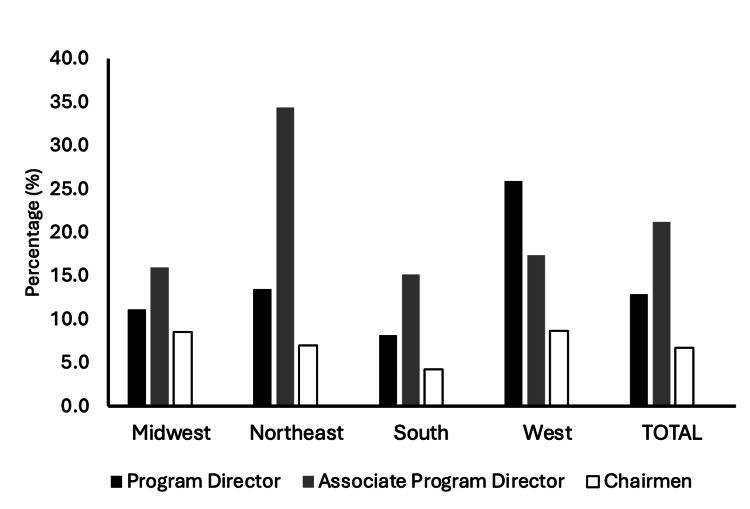
Regional representation of female orthopedic faculty across leadership positions including residency program director, associate program director, and department chair.

A total of 25 (12.9%) female orthopedic residency program directors, and 24 (21.2%) female associate program directors. There were 10 female department chairmen, constituting 6.8% of all department chairs across the United States. When broken down by geographic region, the Midwest region included 54 programs, with 6 (11.1%) having female program directors. In the Northeast, 52 programs were analyzed, with 7 (13.5%) led by female program directors. The South had 61 programs, with only 5 (8.2%) female program directors. The West had the highest proportion of female program directors at 7 (25.9%) (Figure 4).

There was no significant relationship between region and female program director (χ² (6, N =198,) = 6.52; p = 0.368) or region and female chair (χ² (6, N = 148) = 7.08, p = 0.313). However, there was a significant relationship between region and female associate program director (χ² (6, N =113) = 17.34; p = 0.008). The Northeast had the greatest percent per region with a female associate program director at 34.4%.

## Discussion

The present study revealed notable regional variations in orthopedic surgery. In summary, 55 (28%) Midwest, 53 (27%) Northeast, 63 (32%) South, and 27 (14%) West orthopedic surgery residency programs were included. Female orthopedic surgery residents comprised 23.7% of all orthopedic residents across the United States. When analyzed by geographic region, female residents comprised 22.7% of all residents in the Midwest, 24.3% in the Northeast, 22.5% in the South, and 27.0% in the West. In the Northeast region, there was a notable upward trend in the percentage of female residents across PGY5 to PGY1 years (τ = 1.0, p = 0.027). A total of 25 (12.6%) programs had a section in their residency program or orthopedic department homepage website that mentioned and/or was dedicated to women or females. There was significant association between region and homepage mention of women (χ² (8, N = 198) = 105.7; p < 0.001). Overall, 12.9% of all orthopedic residency program directors were female, while 21.2% of all associate program directors were female.

Across the regions, the mean percentage of female residents varied, with the highest representation observed in the West (27.0%), followed by the Northeast (24.3%), Midwest (22.7%), and South (22.5%). These findings differ from existing literature highlighting disparities in gender representation across different geographic areas, underscoring the multifaceted trends observed among female orthopedic surgeons, specifically in academia [8].

However, when analyzing from a class-by-class basis, we observed a notable upward trend in the percentage of female residents across PGY5 to PGY1 years in the Northeast region, (τ = 1, p = 0.027). Additionally, the South and West regions exhibited a strong positive trend in female resident percentages, yet these trends did not reach statistical significance (both τ = 0.8, p = 0.086). The significant upward trajectory suggests progressive strides toward gender parity and may reflect broader societal shifts in attitudes toward gender roles and professional opportunities [9].

Conversely, in the Midwest, although there was a positive trend in the number of female residents from PGY5 to PGY1, it exhibited the weakest relationship strength, supporting that this region may benefit from initiatives aimed at fostering gender diversity in orthopedic surgery. The observed regional disparities in female representation, particularly in the Midwest, may be influenced by several cultural, institutional, and systemic factors. Historically, the Midwest has had fewer urban academic medical centers compared to regions such as the Northeast, where larger institutions often have more established DEI initiatives. The lack of significant trends underscores the need for targeted interventions tailored to regional contexts. One study surveyed 556 members of the Ruth Jackson Orthopaedic Society and found that the most common reasons cited for women not choosing orthopedic surgery included perceived inability to have a good work-life balance, the perception that too much physical strength is required, and lack of strong mentorship in medical school or earlier [5]. Only 27% of respondents acknowledged mentorship as one of their top five influences for pursuing orthopedics. An additional hypothesis for the weaker trend of female orthopedic surgery residents over the years in the Midwest most likely begins at the medical student level. One study observed that gender bias is particularly apparent for women applying to orthopedics, as they are more frequently subjected to illegal interview questions compared to men. For example, family planning questions were posed to 61% of women compared to only 8% of men [10]. One hypothesis explaining the variance in the trend of female orthopedic specialists across regions relates to the policies and practices within medical schools. The Midwest has historically been known to cling to traditional gender norms, often reflecting these values in their curriculum and practices. For example, one study found that sexual and gender minority medical students were less likely to report gender in the central region when compared to the Northeast [11]. This adherence to conventional gender standards may influence various aspects of medical education and training within these regions, leading to decreasing female interest and participation in orthopedic surgery.

Increasing the inclusion of women in orthopedic surgery is a positive step forward. Given that the internet and researching residency programs have become the primary means of accessing information, the study was expanded to evaluate whether orthopedic residency webpages promote inclusivity for women. The present study found that 25 (12.6%) programs featured a section on their residency program or orthopedic department homepage dedicated to women with a significant association between region and the presence of these sections (χ² (8, N = 198) = 105.7; p < 0.001). The largest regional percentage was in the Northeast, with 20.8% of programs featuring a section on their residency program or orthopedic department homepage dedicated to women; the lowest regional percentage was the Midwest at 7.3%. This indicates that regional variations in the inclusion of content related to women may significantly impact female interest in orthopedics. As previously mentioned, the Northeast showed the most pronounced positive trend in female orthopedic residents, whereas the Midwest exhibited the weakest trend. Although analyzing the homepage provides only a snapshot of factors influencing applicant interest, it contributes to reinforcing the observed trends. Highlighting women on orthopedic residency websites could potentially encourage more women to pursue careers in orthopedics.

Additionally, 12.9% of all orthopedic residency program directors were female, indicating a relatively low representation of women in the highest leadership positions. In contrast, 21.2% of all associate program directors were female, indicating a higher representation of women in these secondary leadership roles. Previous studies align with these findings, showing that female orthopedic fellowship program directors are more often assistant professors compared to their male counterparts (39% vs. 22%) [6]. There was a significant relationship between region and female associate program director (χ² (6, N = 113) = 17.34; p = 0.008). specifically, the largest regional percentage was in the Northeast, with 34.4% of programs having a female associate program director. These findings underscore the need for increased efforts to promote gender diversity, particularly in senior leadership positions, and suggest that regional practices and website content might play a role in attracting more women to the field of orthopedics.

Overall, our findings underscore the importance of monitoring and addressing regional disparities in gender representation within orthopedic surgery residency programs. Previous efforts to enhance mentorship and participation of females in orthopedic surgery led to the creation of the Ruth Jackson Orthopaedic Society [12]. As of 2021, the organization boasts 1,161 members, emphasizing mentorship and scholarship, and offering numerous opportunities for members to excel in orthopedics. Additionally, there are other organizations such as the Perry Initiative, a non-profit dedicated to recruiting and retaining women in orthopedics, which has conducted the Medical Student Outreach Program (MSOP) since 2012 [7]. This program offers extracurricular exposure and mentoring to first- and second-year female medical students nationwide, reaching over 300 students in its first three years (2012-2014). One study suggested that the Perry Initiative’s MSOP positively influences women to choose orthopedic surgery, with alumnae matching at twice the rate of females in current residency classes [7]. By identifying trends and disparities, healthcare institutions and policymakers can implement targeted interventions to promote gender equity and cultivate a diverse and inclusive workforce.

There are several limitations of the study. First, the study relied on publicly available information from the websites of orthopedic surgery residency programs. Programs without accessible resident or faculty information were excluded, which may introduce selection bias. Additionally, the accuracy and completeness of the data collected from program websites are uncertain. There is a possibility of selection bias due to reliance on program websites. Programs that actively promote diversity might present more favorable data, while those with less focus on gender equity might not highlight it as prominently. Websites may not be regularly updated, leading to outdated or incomplete information being used in the analysis. Additionally, the study appears to focus on current data without examining longitudinal trends over a longer period, which would help understand the persistence or changes in gender representation over time. Lastly, the categorization of roles (e.g., program director, assistant program director, chair) may vary between programs, leading to potential misclassification or inconsistencies in the data. Future research should emphasize the exploration of regional policies, mentorship structures, and cultural attitudes that impact female participation in orthopedics. Additionally, incorporating qualitative data or institutional surveys could offer deeper insights and contribute to a more comprehensive understanding of these influencing factors. However, despite these limitations, this study provides valuable insights into the regional variations and trends in female representation within orthopedic surgery residency programs, contributing significantly to the ongoing efforts to promote gender diversity in the field.

## Conclusions

This study aimed to provide an updated analysis of the current trends, incorporating a regional perspective on female representation in both orthopedic residency and faculty positions. While progress has been made, significant variation exists in female representation at both the resident and leadership levels. The presence of program-specific initiatives addressing gender diversity remains limited, suggesting an opportunity for institutions to take a more active role in fostering inclusivity. Addressing these disparities requires a multifaceted approach, including mentorship programs, institutional support, and policy-driven efforts to enhance recruitment and retention of women in orthopedics. Additionally, programs can implement structured sponsorship opportunities to support female trainees and foster career advancement. Policymakers and accrediting bodies may also consider incentivizing diversity-focused initiatives through funding opportunities or accreditation standards. Continued monitoring of these trends will be essential in shaping a more equitable and diverse orthopedic workforce. These findings underscore the importance of monitoring and addressing regional disparities in gender representation within orthopedic surgery residency programs. By identifying trends and disparities, healthcare institutions and policymakers can implement targeted interventions to promote gender equity and cultivate a diverse and inclusive workforce.
